# Local knowledge as the basis of disaster management and humanitarian assistance

**DOI:** 10.1111/disa.12634

**Published:** 2024-06-18

**Authors:** Miwa Hirono, Muhammad Riza Nurdin

**Affiliations:** ^1^ College of Global Liberal Arts, Ritsumeikan University Japan; ^2^ Asia‐Japan Research Institute, Ritsumeikan University Japan

**Keywords:** adaptation to new ideas, contextual historical memories, disaster management, humanitarian assistance, local knowledge, localisation, locally‐led, social capital

## Abstract

Recent policy discourse on the localisation of disaster management and humanitarian assistance lacks attention to the culture, history, and traditions of the Global South. This special issue of *Disasters* argues that it is imperative to recognise the dynamic, interactive, contested, and negotiated nature of local knowledge. Such local knowledge saves lives by enabling responders to situate ad hoc, one‐off events such as disasters in the broader and deeper context of community relationships, thereby providing more appropriate and more effective aid. Through the cases of China, Japan, Indonesia, and the Philippines, this special issue examines such dynamic local knowledge using an analytical framework consisting of three manifestations of local knowledge, namely: social capital; contextual historical memories; and adaptation to new ideas. These three manifestations show the ways in which local knowledge creates local capacity, via which local, national, and international disaster respondents can centre their response coordination, and in turn, demonstrate how local capacity reformulates local knowledge.

## 要旨

災害管理と人道支援の現地化に関する近年の政策議論には、アジア諸国を含むグローバル・サウスの文化、歴史、伝統への配慮が欠けている。本特集論文では、災害管理と人道支援のサイクルのすべての要素を分析する際に、ローカルナレッジの対話的でダイナミック、論争的、交渉的な性質を認識することが不可欠であることに関して議論する。これにあたり、本特集論文では、こうしたダイナミックなローカルナレッジを、社会資本、文脈記憶、新しいアイデアへの適応というローカルナレッジの3つの具現型を通じて包括的に検討する。これら3つの具現型は、ローカルナレッジが地域の力を生み出し、それをもとに地域、国、そして国際的な災害対応者がどのように対応調整を行うか、そして逆に、地域の力がローカルナレッジをどのように再形成するかを示している。

## 摘要

最近关于灾害管理和人道主义援助本地化的政策讨论缺乏对包括亚洲国家在内的全球南方国家的文化、历史和传统的关注。本期特刊指出，在分析灾害管理和人道主义援助周期的所有要素时，必须认识到当地知识的互动性、动态性、争议性和协商性。为此，本特刊通过地方知识的三种表现形式，即社会资本、情境记忆和对新思想的适应，对这种动态的地方知识进行了全面研究。这三种表现形式展示了地方知识如何创造地方能力，地方、国家和国际灾害应对人员可以以此为中心进行响应协调；反过来，也展示了地方能力如何重新形成地方知识。

## 1 INTRODUCTION

The ‘localisation’ agenda features global policy and academic discourse on disaster management and humanitarian assistance. The Sendai Framework for Disaster Risk Reduction 2015–2030 and the Grand Bargain agreement of 2016 both emphasise the importance of the localisation of disaster management and humanitarian assistance. The Grand Bargain's ‘Commitments on Localisation’, for example, refer to financial and institutional arrangements used to strengthen ‘the institutional capacities of local and national responders’ (International Federation of Red Cross and Red Crescent Societies, 2018). Pacific researchers place more emphasis on the context of the Pacific Islands, and define localisation as ‘a process of recognising, respecting and strengthening the independence of leadership and decision making by national actors in humanitarian action, in order to better address the needs of affected populations’ (Ayobi et al., 2017, p. 4). Localisation is also a part of broader development debates, as indicated in various policy documents such as the Paris Declaration on Aid Effectiveness (2005), the Busan Partnership for Effective Development Co‐operation (2011), the 2030 Sustainable Development Agenda (2015), the OECD–DAC (Organisation for Economic Co‐operation and Development–Development Assistance Committee) Recommendation on Enabling Civil Society in Development Co‐operation and Humanitarian Assistance (2021), and the Principles for Locally led Adaptation (2021). While approaches to localisation vary, what is in common in the discourse of localisation is critical reflection on the fact that Western states and organisations have long dominated the international humanitarian policy discussions. Localisation is an effort to change such domination by encouraging local actors to retain leadership of, or involvement in, humanitarian efforts.

One fundamental, yet often neglected, problem with the localisation agenda, however, is that global policies do not necessarily reflect what really matters to the people in disaster‐affected local communities—what we call ‘local knowledge’. As elaborated later, local knowledge is defined as a process of negotiating contested understandings of how a community works; which community members gain through their history and their experience of disasters. It is culturally, historically, and traditionally situated, and is never static but rather dynamic, evolving constantly. Another reality is that when disasters hit, local communities often seek help from non‐ or quasi‐institutionalised actors, such as family members near and far, neighbours, the diaspora, religious groups, children's school communities, and, today, more commonly, digital communities (fundraising through Facebook, for example). The policy community, though, assumes that local actors seek help from institutionalised actors, such as local governments and non‐governmental organisations (NGOs). Nor does it accord more than scant attention to the importance of local knowledge. An extension of such assumptions is that when international policy attempts to promote capacity‐building in disaster management, it pays most attention to institutional and technological capacity, but conspicuous by its absence is due regard to the rich capacity and resources available in many disaster‐prone regions—local knowledge—which are frequently held by local and non‐ or quasi‐institutionalised actors. This special issue of *Disasters* aims to address the problem by asking how local knowledge saves lives and makes all elements of the cycle of disaster response more effective.

The international aid community has begun to recognise the importance of local knowledge and to make efforts to include local knowledge in its work. The international agreements mentioned earlier—especially the Sustainable Development Goals and the Principles for Locally led Adaptation—demonstrate this by mentioning local knowledge as a part of resilience. Some agencies also try to draw on local knowledge as part of their daily work. The United States Agency for International Development (USAID), for instance, has developed rich resources to assess local knowledge for its practical use. It offers a robust ‘toolbox’ to ‘identify, value, and apply local knowledge’ (USAID, n.d., p. 1), and encourages USAID staff and partners to utilise this ‘toolbox’ in their engagement with a variety of stakeholders in all programme cycles. Specifically, it suggests increasing community participation in evaluating ongoing or completed development projects, identifying gaps between marginalised groups and general populations, examining the multiple dimensions of power (including cultural, gender, political, religious, and social), and pinpointing any potential negative effects of development activity on local communities (USAID, n.d.).

While these efforts would advance the quality of aid projects by engaging with a wide range of stakeholders in a more inclusive way, they may risk objectifying ‘local knowledge’—treating it as a ‘thing’. Local knowledge is neither static nor uniform throughout the development of a community. In other words, it is not like a roll cake, the inside of which has the same consistency from edge to edge, so that wherever you slice it, the same inner surface appears. Local knowledge is constantly evolving; comprehending it from the inside out (not explaining ‘something’ from the outside in) requires recognition of local knowledge as dynamic, contested, and negotiated. Ensuring that response coordination centre local knowledge requires, as a first step, recognition of local knowledge as it is—that is, going beyond its static image to understand and embrace its dynamic, contested, and negotiated nature. Such recognition has potentially wide significance with respect to policy change.

This special issue aims to do just that. The papers included in this collection investigate what such knowledge looks like in specific case studies, and what difference it makes or has made to the lives of people in disaster‐affected communities. This special issue establishes an analytical framework with which to explore how local knowledge is generated, reformulated, or sometimes forgotten on the ground.

Knowledge is a somewhat abstract concept. By focusing on empirical phenomena in which knowledge manifests in concrete cases of disaster response we are able to identify what local knowledge is and how critically important it is to any response to a disaster. Overall, this special issue argues that local knowledge saves lives by enabling the responders to situate ad hoc, one‐off events such as disasters in the broader and deeper context of community relationships, thereby providing more appropriate and more effective aid. It provides ‘frames of meaning’ which reorient disaster responses and help to break down the divide between action and culture, and streamline disaster response by affording it broader, multiple, and overlapping contexts.^1^ Such local knowledge is reidentified, negotiated, and developed by appropriating what seems to be ‘unrelated to disaster response’ and directing it to a more nuanced overall disaster response. This special issue advocates not only changes in practice but also changes in policy, thus ‘centring’ local knowledge in a more comprehensive and better coordinated response.

This introductory paper is divided into four sections. After discussing the proposed definition of local knowledge in the next section, the third section appraises why it matters to disaster response today. The fourth section points out the three manifestations of local knowledge that comprise the analytical framework of this special issue; this can be used by disasters practitioners in disaster‐affected areas to identify hidden local knowledge and centre it in their work. The fifth section introduces each paper included in this special issue and looks at how each submission advances our understanding of local knowledge as the basis of disaster management and humanitarian assistance.

## 2 DEFINITION OF LOCAL KNOWLEDGE

This special issue puts forward the following working definition of local knowledge:

a *process* of negotiating contested understandings of how a community works in disasters; which community members gain through *social practice* in everyday life. It is situated in a cultural, historical, social, and traditional *context*, and is never static but constantly and dynamically evolving.

The emphasis on a *process* through *social practice* derives from an interpretivist approach to anthropology, as represented by Clifford Geertz's work. ‘Local knowledge’ became a popular but contentious concept when Geertz (1983) published his book titled *Local Knowledge: Further Essays in Interpretive Anthropology*. In it, he advocated that culture is not something to be explained; it is a *process* in which meaning is constantly interpreted. Culture, according to Geertz, should be understood as ‘the “webs of meaning” within which people live, meaning encoded in symbolic forms (language, artifacts, etiquette, rituals, calendars, and so on) that must be understood through acts of interpretation analogous to the work of literary critics’ (Ortner, 1999, p. 3).

Local knowledge is a process of negotiating contested understandings through *social practice*. It is ‘not a philosophical paradigm or a body of ideas’ (Canagarajah, 2002, p. 251). Rather, ‘celebrating local knowledge refers to adopting a practice’ (Canagarajah, 2002, p. 251). In other words, local knowledge does not amount to traditional beliefs per se. Local knowledge is ‘a process of negotiating dominant discourses and engaging in an ongoing construction of relevant knowledge in the context of our history and social *practice*’ (Canagarajah, 2002, p. 251; emphasis added).

Discussion of this definition is based on a critical view of the dichotomous understanding of knowledge (Agrawal, 1995). Western, modern, and scientific knowledge was often linked to universality and civilisation and regarded as something superior to outdated and backward non‐Western, traditional, and indigenous knowledge. In the 1980s and 1990s, some scholars in development studies problematised this form of cultural imperialism (Kothari, 1985; Agrawal, 1995), while others criticised this as a ‘cultural trap’ and argued that those who criticise cultural imperialism in development ‘romanticise indigenousness and wrongly blame the west for pitfalls of industrialism’ (Sarkar, 1995, p. 1846). If development is rejected, Sarkar (1995, p. 1846) asserts, it should be rejected only when it is ‘ecologically, economically, politically and socially impossible and/or harmful’, not when it does not address indigenous culture.

While this debate helped the development literature evolve further with regard to how we understand knowledge, both sides of the debate based their argument on a dichotomous view of knowledge. In their thinking, modern knowledge and traditional knowledge are distinct *entities* that have some degree of closure and boundary, which need to be defined and differentiated from each other.

Instead, current studies taking a pluralist approach go beyond such an understanding to discuss the process via which such knowledge is created. Local knowledge should be comprehended as an evolving process comprising modern and traditional practices. In the daily life of many societies, these practices are not necessarily in a binary relationship but rather are complementary. As Suresh Canagarajah (2002, p. 244) claims, ‘local knowledge is context bound, community specific, and nonsystematic because it is generated ground up through social practice in everyday life’.

Practices evolve in a particular social, cultural, historical, and traditional *context*. One of the more important contexts is a social hierarchy determined by different structures such as ‘class, gender, ethnicity, age, disability and immigration status’ (Wisner et al., 2004, p. 8). Community members located at the bottom of social hierarchy, such as women, elders, people with disabilities, minorities, and other marginalised groups, are often left out of practices of disaster management, creating vulnerabilities and exposure to hazards. Another context is enshrined in local cultural beliefs, often associated with indigenous and religious interpretations of disasters. Disasters are considered by many communities to be acts of, or the will of, God (Steinberg, 2006; Lutzer, 2011; Park, 2016; Rahiem, Abdullah, and Krauss, 2017; Aksa, 2020). While this perception of disaster fatalism needs to be changed, scholars agree that religious beliefs and practices should be respected (Chester, 2005) and recognised as one of several coping strategies after a disaster (Ekanayake et al., 2013; Fletcher et al., 2013; Gianisa and Le De, 2017; Samuels, 2019). Effective disaster management practices require attention to these various contexts to help a community coping with a disaster.

## 3 WHY LOCAL KNOWLEDGE MATTERS TO DISASTER RESPONSE

There are practical and ethical reasons why it is pertinent to examine how local knowledge saves lives and makes all elements of the cycle of disaster response more effective.

The practical reason to focus on local knowledge is that it has great potential to save lives. In all walks of life, it is imperative to know the field in which one operates. Disaster respondents need to contend with not only the nature of disasters and material damage to lifelines and infrastructure, but also how a community's members relate to each other, and to non‐community actors such as governments, external civil society entities, and businesses. Such relationships are embedded in the community's history, culture, and tradition. Irrespective of whether the respondents themselves are community members or outsiders, local knowledge in this context can be a critical response capacity, something which all disaster response practitioners must have in their repertoire. Such knowledge offers us contextual appropriateness that can help in the process of tailoring interventions to the community's unique needs in the short and longer term. It will therefore enhance the likelihood of its effectiveness in the short term, and resilience and sustainability in the longer term. Furthermore, in the building of communication and trust between responders and the community receiving aid, local knowledge plays a crucial role in elucidating a shared understanding of the emergency and what sort of response is necessary. In this regard, understanding of local language amounts to local response capacity, too—a capacity that goes beyond knowing simply the words to say, to also knowing the ways in which to say these words, and the importance of their contextual meaning. It is imperative that response coordination be centred on local knowledge as much as possible in developing an historically and culturally embedded disaster response. A disaster‐affected community has a wealth of intellectual resources and capacity, most of which are not necessarily recognised in the current sense of response coordination.

The ethical reason to focus on local knowledge has to do with the need to go beyond neocolonialism and the ‘victimisation’ of disaster respondents, which were still highly emphasised during the Grand Bargain negotiation of 2016. The paternalism of the current aid system that often depicts local actors as passive ‘victims’ of disaster, rather than as proactive ‘respondents’, overlooks the fact that many non‐Western disaster‐prone regions have thousands of years of history and traditions of relevance vis‐à‐vis responding to disaster. During their long histories, such regions have accumulated very rich knowledge and experience of disaster response. They exhibited expertise and autonomy in disaster response well before the modernisation period. Such expertise has been undeservingly underestimated owing to a power imbalance between Western traditional donors and non‐Western regions. Locals are the central disaster respondents deciding their own affairs by themselves; their knowledge and experience should be at the centre of disaster response efforts. It is important to pay attention to the primacy of the local contexts in which humanitarian and disaster responses take place.

Yet, one might question the validity of local knowledge. In what way does ‘local’ play an important role in effective disaster management and humanitarian assistance? Despite agreement on the importance of local people, there is not enough academic research as to when local matters, and when national or international matters (Howitt and Leonard, 2009, p. 4). Relatedly, what if particular local knowledge is inappropriate or just simply wrong. There are cases where non‐local knowledge—global or national, for example—matters more to disaster response than local knowledge (see the paper by Caroline Reeves in this special issue). Rather than romanticising local knowledge, it is important to look for empirical cases of when and how local knowledge saves lives. Many papers in this special issue demonstrate that with concrete case studies.

## 4 ANALYTICAL FRAMEWORK

To establish how local knowledge manifests in real‐world situations and how it makes a difference to the lives of people in disasters, this special issue proposes an analytical framework to identify manifested forms of local knowledge and suggest models for how each manifestation helps to save lives or contributes to effective disaster governance. As noted, there are three types of local knowledge that can be useful for disaster management and humanitarian assistance: social capital; contextual historical memories; and adaptation to new ideas. In the case studies in this special issue, each of these types of knowledge, constructed and negotiated over time, is of central importance to various local actors themselves or to external actors to make the locally‐led approach to disaster management and humanitarian assistance efficient.

This analytical framework helps not only scholars to identify and analyse local knowledge, but also respondents to re‐examine their own locality and recognise their existing locally‐led capacity for disaster management. To spot capacity gaps, the identification of existing local capacity, taking fully into consideration the dynamic nature of local knowledge, is crucial. The following subsections explain each type of local knowledge and how they can be useful for disaster management and humanitarian assistance.

### 4.1 Social capital

Social capital is an important concept in disaster management and humanitarian assistance. Francis Fukuyama (2000, p. 1) states that social capital is ‘an instantiated informal norm that promotes cooperation between individuals’. As such, it is based on human relations and reciprocity to produce a cooperative end, which in this context is to save lives and/or formulate effective disaster governance. Norms, which are derived from local knowledge such as religious values and cultural virtues, play a crucial part in creating elements of social capital, namely, social networks, trust, and bonds.

We argue, therefore, that the relationship of social capital to local knowledge is inextricably linked, with both elements being mutually interconnected. Local knowledge is important in building effective social capital. Existing studies have demonstrated that local knowledge is central to the mobilisation of social capital (Nakamura and Kanemasu, 2020); good use of the former can make the latter a ‘force multiplier’ (Aldrich, 2012, p. 174). Local knowledge is also essential to preserving a community's social capital (Hiwasaki et al., 2014). Yet, social capital is crucial to the growth and propagation of local knowledge. Scholars assert that it can be a driver of local knowledge exchange (Díez‐Vial and Montoro‐Sánchez, 2014), facilitator of the transfer and evolution of local knowledge (Dasanayaka and Matsuda, 2022), and creator of new knowledge (Nieves and Osorio, 2015; Sheng and Hartono, 2015). This last function, however, has been discussed mainly outside the area of disaster studies, leaving a gap in disaster studies that this special issue attempts to fill.

A typical example of social capital is a community gathering in and around religious events such as prayer sessions, where community members share information and apply local knowledge of how community affairs unfold, thus strengthening social capital within a community. As Muhammad Riza Nurdin's paper in this special issue demonstrates, an NGO staff member put local knowledge into practice by developing a close relationship with the youth leader of a village in central Aceh in Indonesia—who is also a member of the most influential family in the village—and by making frequent visits to the village mosque to disseminate information about the disaster recovery programme. The application of local knowledge helped the village member to strengthen his internal bond in the community—in other words, to build social capital among community members.

To centre their work on the social capital aspect of the community, disaster respondents should be asking questions including: who are the highly effective people in the community who either lead or lubricate the process of human interaction, not only within the community but also with other communities, or even with external donors? What are the key areas in which such people can enhance the social capital of the community, in relation to bonding within the community, and bridging and linking the community and outsiders?

### 4.2 Contextual historical memories

Every community has historical memory that has developed over time, which enables community members to share a particular interpretation of the meaning of a community activity, including disaster management and humanitarian assistance. Disaster respondents must consult with this type of local knowledge so that their action is properly understood by the members of a recipient community. Historical memory is defined as ‘the collective understandings that a specific group shares about events in the past that it perceives to have shaped its current economic, social, cultural, and political status and identity’ (Davis, 2005, p, 4). Such memory is critically important to the actions taken in disaster management and humanitarian assistance because it provides the *context* in which an action is taken—hence, we call this frame ‘contextual historical memories’. Contextual historical memories afford a better empirical basis on which to assess whether a particular action has been helpful to local people at the receiving end of the action (Fierke, 2007).

Contextual historical memories matter to our investigation because they are a form of local knowledge that response coordination must spotlight in its work, especially when a particular locality has some conflictual relationships within the area or with an outsider. Imagine a locality that has an ethnic minority group that has been discriminated against by the majority group. The ethnic minority group and the majority group respectively have their own historical memories, which provide the contexts within which disaster respondents must work. Contextual historical memories as local knowledge are critically important to deciding which group gets what type of assistance, and in what order. If humanitarian assistance is provided to the minority group without considering the local context, such aid will likely be opposed by the majority group, which may obstruct its smooth distribution. Many scholars and practitioners have claimed that a conflict‐sensitive approach is vitally important to an outsider's intervention in conflict (Anderson, 1999; Conflict Sensitivity Consortium, 2012; Inter‐Agency Standing Committee, 2020). The same can be said of disasters triggered by a natural hazard. A conflict‐sensitive approach is only possible when contextual historical memories are available to the disaster respondents. Racial and ethnic discrimination between communities is a case in point. When assistance is provided to a community with discrimination or social hierarchy vis‐à‐vis ethnic groups or gender, what the outsider does can be sensitive—whether one should go along with the existing social structure to prioritise stability or introduce a condition of equal and inclusive distribution of assistance. The reality is not always a dichotomy. A lot of dialogue is involved to find an intricate balance between the needs of various stakeholders. The first step towards such dialogue is to comprehend the contextual historical memories.

Disaster respondents should be asking questions including: have they themselves understood the sociocultural contexts of the community? Have they comprehended local contexts within the community, for example, where there is an ethnic divide, a social hierarchy, or prejudice?

### 4.3 Adaptation to new ideas

Some communities have historically developed approaches in which they manage their relationships with outsiders who come with different values and beliefs. Miwa Hirono (2008) claims that there are three types of interaction with outsiders: assimilation; conflict; and new consciousness. Assimilation takes place when a community changes itself to accept values and beliefs deriving from outside. This can occur when a powerful outsider forces a community to change or when a community ends up changing itself to conform to alternative values and beliefs for some reason. The opposite type of interaction is conflict. This manifests when different values and beliefs clash, and a community refuses to change its values and beliefs. In between the two could be a partial adaptation of different values. Interaction between different communities sometimes leads to neither conflict nor assimilation, but to ‘something new’ or ‘something unexpected’. This is not something between assimilation and conflict; rather, ‘it refers to a more mature and deeper relationship between the two parties, which eventually leads to unexpected consequences at the beginning of the interaction’ (Hirono, 2008, p. 36). Hirono calls it a ‘new consciousness’, which goes beyond the dichotomous understanding of the relationship between the two parties. It refers to a situation in which a community seizes the interaction with outsiders as an opportunity to change its self‐consciousness by itself. As a result of interaction, ‘community identity is likely to be changed or reinforced significantly as a result of self‐realisation’ (Hirono, 2008, p. 40). The idea of permaculture, reconfirming how cultural practice helps one mitigate disasters, resonates with this type of relationship. For instance, the interaction of some communities in Bali, Indonesia, with modernising forces from outside led them to develop a permaculture approach, which takes into account their historically developed ecological living and reconfirm their way of living, while incorporating some modern technology to farming (IDEP Foundation, n.d.).

This frame relates to how a particular community engages with outsiders. It classifies the community's preferred model of interaction as a part of their local knowledge. Knowing how the particular community has engaged historically with outside communities is important in engaging with non‐local communities—whether they are donors, a national government, or neighbouring communities. Depending on whether a particular community tends to take an assimilative or conflictual approach to other communities will give one an important context in which disasters need to be managed or humanitarian assistance delivered. Misunderstandings can hamper effective engagement with a community, which may cost lives. Whether the community has gone through the frame of new consciousness and adapted to new ideas is also important when outsiders approach the community with disaster management and humanitarian assistance. This knowledge will provide overall direction with respect to the issue of how outsiders can take into account the cultural practices and identity of the target community.

Respondents should be asking questions including: what has been the community's experience of working with external stakeholders such as donors, international organisations, the government, and NGOs? Have we identified how the local community tends to assimilate outside values and knowledge, resist them, and/or create some new identity or new practice?

## 5 THE STRUCTURE OF THE SPECIAL ISSUE

This special issue argues that it is imperative to recognise the interactive, dynamic, contested, and negotiated nature of local knowledge in analysing all elements of the cycle of disaster management and humanitarian assistance. In so doing, it comprehensively examines such dynamic local knowledge through the aforementioned three manifestations of local knowledge, namely: social capital; contextual memories; and adaptation to new ideas. These three manifestations show the ways in which local knowledge creates local capacity, through which local, national, and international disaster respondents can centre their response coordination; and in turn, the local capacity reformulates local knowledge. The papers in this special issue approach the manifestations of local knowledge in a multidisciplinary way, bringing together specialists in anthropology, computer science, conflict management, development studies, gender studies, history, international relations, and marine biology, all of whom approach the same questions regarding what local knowledge is, why local knowledge matters, and how local knowledge saves lives and mitigates disasters. We draw on the experiences of disaster and humanitarian actors in China, Indonesia, Japan, and the Philippines—all disaster‐prone regions where we can clearly observe local knowledge as an evolving process through social practices in everyday life.

Two papers focus on the process of formation of social capital as a way to demonstrate one of the processes in which local knowledge evolves. Muhammad Riza Nurdin's paper investigates the role of Islamic faith‐based organisations (FBOs) and their social capital formation in post‐disaster Indonesia, specifically the 2013 earthquake in Aceh and the 2014 Mount Kelud volcanic eruptions in East Java. His paper assesses the extent to which FBOs are able to formulate social capital when delivering disaster recovery assistance to affected communities in Indonesia, and the conditions under which the social capital formulation process can have positive or negative consequences for communities affected by disaster. This paper shows precisely a process of negotiating contested understandings of how a community works in disasters, which community members gain through social practice in everyday life (the definition of local knowledge in this special issue); the result of such a process is the generation of social capital, or a failure to do so.

This is followed by a case study by Yasuko Hassall Kobayashi, which concentrates on how government institutions can play a key role in formulating social capital across migrant communities, thereby safeguarding the lives of migrants in times of disaster. Her paper focuses on the 2018 northern Osaka earthquake and contributes to the discussion of evolving local knowledge by paying attention to social capital building and two blind spots in the literature: the role of government (instead of civil society actors) in formulating social capital; and the community of new migrants, which have not created any social capital. Our definition of ‘local knowledge’ began with the assumption that there are contested understandings of how a community works in disasters, but her analysis in fact commences from a situation in which no community of migrants exists. It must be built from the ground up. Nonetheless, Minoh City in northern Osaka managed to build up social capital prior to and during the earthquake. Her paper discusses how this can be done.

The next two papers pay particular attention to an aspect of local knowledge: contextual historical memories. Maria Tanyag argues that local knowledge of ‘forgotten crises’ is a valuable resource for conflict and disaster prevention. By focusing on some examples in the Philippines when conflicts, disasters, and pandemics interact in the context of climate change, her contribution problematises ‘crisis competition’—when a number of crises unfold in multiple layers simultaneously, a less visible crisis is easily forgotten. Instead, visible crises that are often associated with urgency and supported by the powerful in society are given priority in the response. Her paper asserts, however, that local knowledge, in the form of contextual historical memories, can serve as a valuable resource for bridging together conflict and disaster prevention by simultaneously addressing the root causes of conflicts, disasters, and poverty.

Miwa Hirono's paper on the role of the diaspora in international rescue and reconstruction efforts also highlights the importance of contextual historical memories. While the diaspora is a local community that is overlooked by traditional donors, it can play a significant role in formulating social capital between communities and between a community and disaster respondents coming from outside. The paper centres on the relationship between the Chinese diaspora in Aceh, Indonesia, and the People's Republic of China's (PRC) disaster respondents in the aftermath of the Indian Ocean tsunami in 2004. The Chinese diaspora in Aceh supported the PRC's international rescue team with language and logistics, but more importantly, it offered contextual historical memories by which major PRC actors, such as the Chinese rescue team, the Red Cross Society of China, and the China Charity Foundation, were able to operate in the province without causing political concerns.

The third manifestation of local knowledge, adaptation to new ideas, is represented by two case studies. One is historical cases of plague in early twentieth century China. While this special issue began by questioning the superiority of scientific knowledge, Caroline Reeves deals with a fact that needs to be recognised: that there is ‘superior’ knowledge that can save lives in a disaster situation, such as in a pandemic. Some fundamental questions in her historical cases are quite contentious, dealing with how some communities have overlooked the importance of science in today's COVID‐19 (Coronavirus disease 2019) discussion. She asks what happens when there is ‘superior’ knowledge held by the non‐local community which is not available and/or accepted at the local level, but which can save lives in a disaster? How can non‐local expertise be integrated into local cultural contexts? In public health emergencies, what is the correct balance between safeguarding autonomy and saving lives? Her paper points up the complexities of the relationship between global knowledge and local acceptance of that knowledge, the variation in what constitutes effective ‘local’ cooperation in adopting international knowledge, and the critical importance of the locality to the landscape of disaster response.

While Caroline Reeve's paper assumes the superiority of a particular international knowledge and local cooperation in adopting it, the paper by Gian Powell B. Marquez and Ronald Dionnie Olavides identifies ‘hybrid ecological knowledge’ in the case of mangrove conservation and rehabilitation as a solution for disaster risk reduction—another case of adaptation to new ideas. Through community participation, creating hybrid ecological knowledge—the integration of science‐based ecological knowledge into existing local ecological knowledge—helps to facilitate long‐term community‐based mangrove management beyond project duration by empowering the community and enabling project acceptance and ownership. Still, continuous local institutional support is a necessary anchor for community resilience.

The final paper by Daniel Moritz Marutschke, Muhammad Riza Nurdin, and Miwa Hirono is related to social capital, but its main aim is to introduce a methodological innovation, using a language model based on artificial intelligence (AI). Understanding local knowledge requires an in‐depth contextualisation of observed community dynamics and of community members' comments. For that, qualitative content analysis is important. However, analysing complex social phenomena would benefit from revalidation of research results using alternative methods. This paper uses the same data as Muhammad Riza Nurdin's paper in this special issue. It develops an AI‐based language model and utilises it to validate the findings of the earlier qualitative research, as well as gleaning additional insights that the qualitative research might otherwise have missed. An AI‐based language model could therefore be beneficial to other research on the importance of local knowledge to effective disaster response because of its ability to conduct in‐depth contextual analysis quantitatively.

These seven papers together showcase a variety of ways in which local knowledge manifests and creates local capacity, and in turn, demonstrate how local capacity reformulates local knowledge. The authors of this special issue of *Disasters* hope that their papers will help readers identify often neglected local knowledge and local capacity, through which local, national, and international disaster respondents can centre their response coordination, thereby providing more appropriate and more effective disaster management and humanitarian response.

## ETHICS STATEMENT

This paper reports analysis of secondary sources.

## Data Availability

The data that support the findings of this study are available on request from the corresponding author. The data are not publicly available due to privacy or ethical restrictions.

## Endnote

^1^
We draw upon sociological institutionalism in the study of corporate governance in arriving at this argument. See Dexter (2018).
